# Emergence of Nosocomial Pneumonia Caused by Colistin-Resistant *Escherichia coli* in Patients Admitted to Chest Intensive Care Unit

**DOI:** 10.3390/antibiotics10030226

**Published:** 2021-02-24

**Authors:** Mohamed A. El-Mokhtar, Enas Daef, Aliae A. R. Mohamed Hussein, Maiada K. Hashem, Hebatallah M. Hassan

**Affiliations:** 1 Department of Medical Microbiology and Immunology, Faculty of Medicine, Assiut University, Assiut 71515, Egypt; elmokhtarma@aun.edu.eg (M.A.E.-M.); enasdaef@aun.edu.eg (E.D.); 2 Microbiology and Immunology Department, Faculty of Pharmacy, Sphinx University, Assiut 71515, Egypt; 3 Chest Department, Faculty of Medicine, Assiut University, Assiut 71515, Egypt; aliaehussein@aun.edu.eg (A.A.R.M.H.); maiada.hashem@aun.edu.eg (M.K.H.)

**Keywords:** multidrug-resistance, colistin resistance, *mcr-1*, enteroaggregative *E. coli*, ESBL, MBL

## Abstract

(1) Background: Colistin is a last-resort antibiotic used in treating multidrug-resistant Gram-negative infections. The growing emergence of colistin resistance in *Escherichia coli (E. coli)* represents a serious health threat, particularly to intensive care unit (ICU) patients. (2) Methods: In this work, we investigated the emergence of colistin resistance in 140 nosocomial *E. coli* isolated from patients with pneumonia and admitted to the chest ICU over 36 months. Virulence and resistance-related genes and *E. coli* pathotypes in colistin-resistant and colistin-sensitive isolates were determined. (3) Results: Colistin resistance was observed in 21/140 (15%) of the nosocomial *E. coli* isolates. The MIC_50_ of the resistant strains was 4 mg/L, while MIC_90_ was 16 mg/L. Colistin-resistant isolates were also co-resistant to amoxicillin, amoxicillin/clavulanic, aztreonam, ciprofloxacin, and chloramphenicol. The mechanism of colistin resistance was represented by the presence of *mcr-1* in all resistant strains. Respectively, 42.9% and 36.1% of colistin-resistant and colistin-sensitive groups were extended-spectrum β-lactamase (ESBL) producers, while 23.8% and 21% were metallo β-lactamase (MBL) producers. *bla_TEM-type_* was the most frequently detected ESBL gene, while *bla_IMP-type_* was the most common MBL in both groups. Importantly, most resistant strains showed a significantly high prevalence of *astA* (76.2%), *aggR* (76.2%), and *pic* (52.4%) virulence-related genes. Enteroaggregative *E. coli* (76%) was the most frequently detected genotype among the colistin-resistant strains. (4) Conclusion: The high colistin resistance rate observed in *E. coli* strains isolated from patients with nosocomial pneumonia in our university hospital is worrisome. These isolates carry different drug resistance and virulence-related genes. Our results indicate the need for careful monitoring of colistin resistance in our university hospital. Furthermore, infection control policies restricting the unnecessary use of extended-spectrum cephalosporins and carbapenems are necessary.

## 1. Introduction

Multidrug-resistant (MDR) Gram-negative bacteria are among the most serious public health problems worldwide and are particularly important in developing countries where the misuse of antibiotics is highest [1]. The Center for Disease Control and Prevention (CDC) reported that about two million patients are infected with MDR microorganisms in the United States, and about 32,000 Americans die annually [2]. Colistin, also known as polymyxin E, is a positively charged polypeptide antibiotic that was discovered in 1949 and was effective in the treatment of Gram-negative bacterial infections. However, colistin use was clinically limited because of the reported side effects, such as the high incidence of nephro- and neurotoxicity [3]. The development of MDR bacteria and the attractive activity of colistin against these agents have necessitated the reintroduction of this old antibiotic to clinical practice [4]. Nowadays, colistin is one of the last resorts in treating serious, difficult to treat infections caused by the MDR Gram-negative bacteria, including carbapenem-resistant agents [5].

Unfortunately, increasing resistance rates to colistin have been reported worldwide, which is attributed, at least to a great extent, to the clinical misuse of colistin and/or the use of colistin in veterinary medicine. Therefore, active surveillance and monitoring of colistin resistance and the underlying resistance mechanisms are of paramount importance, particularly in patients with critical conditions such as those admitted to intensive care units (ICUs) [6].

Plasmid-mediated colistin-resistance was first described in China and was attributed to the presence of the *mcr-1* gene, which encodes a transferase enzyme that inactivates colistin [7]. The *mcr-1* gene was not only confined to the plasmid but was also reported to be located on the bacterial chromosome [8]. Later on, additional *mcr* genes (*mcr-2* to *mcr-10*) were also described globally [9,10,11,12,13]. Several *mcr-1*-positive *Escherichia coli (E. coli*) have been isolated from both humans and animals in many different countries, which greatly impacted colistin clinical treatment strategies [14,15].

Healthcare-associated infections (nosocomial infections) are known to increase patients’ morbidity and mortality. In ICUs, the rate of nosocomial pneumonia exceeds that in general wards by 5–10 times. Moreover, the incidence of pneumonia among these critically ill patients ranges from 7% to more than 40% [16]. Nosocomial pneumonia in Europe increased the mortality rate by 6%, and duration of stay in the ICU and mechanical ventilation (10–12 days) [17]. The objective of this study was to evaluate colistin resistance in *E. coli* isolated from patients with nosocomial pneumonia, describe the resistance mechanism, and evaluate the associated resistance and virulence-related genes in the isolated colistin-resistant *E. coli* strains.

## 2. Material and Methods

### 2.1. Bacterial Strains

*E. coli* isolates were recovered from respiratory samples that were collected over a period of 36 months (from January 2017 to December 2019) from patients admitted to the chest ICU, Assiut University Hospital, which is one of the main tertiary hospitals in Upper Egypt. *E. coli* isolates were recovered from sputum, bronchial, and tracheal aspirate (in mechanically ventilated) samples of patients suffering from hospital-acquired pneumonia and were phenotypically identified using the API 20E system (Biomérieux, Marcy l’Etoile, France). Hospital-acquired pneumonia was defined as pneumonia that developed 48 h after admission, excluding infections that were incubating upon admission [18].

### 2.2. Sampling Process

Sputum and aspirate samples were collected for the microbiology assessment before starting the empirical antibiotic therapy. For patients who received inadequate empirical antibiotic treatment and still suffered from signs and symptoms of infection, the antibiotic therapy was continued based on the antibiogram results of the isolated pathogen. Inadequate treatment was considered if the initial empirical antibiotic therapy did not contain an effective drug against the isolated microorganism.

Samples were incubated aerobically at 37 °C for 24 h on suitable culture media: blood agar and MacConkey agar. *E. coli* was preliminarily identified by its characteristic growth on the differential media and was further confirmed using the API system (API 20E, BioMérieux, Marcy l’Etoile, France).

### 2.3. Antibiotic Susceptibility Testing

The antibiotic susceptibility profile of the *E. coli* isolates was determined using the Kirby–Bauer disc diffusion technique [19]. The following commonly used antibiotics were tested: amikacin, amoxicillin, amoxicillin/clavulanic acid, aztreonam, cefaclor, ceftriaxone, cephazolin, chloramphenicol, ciprofloxacin, levofloxacin, imipenem, and meropenem. Susceptibility testing was performed by inoculating Mueller–Hinton agar plates (Thermo Fisher Scientific, Waltham, MA, USA) with a bacterial suspension equivalent in turbidity to 0.5 McFarland. Plates were then incubated overnight at 37 °C before recording the results according to the Clinical and Laboratory Standard Institute guidelines [20].

### 2.4. Colistin Susceptibility Test

The use of the disc diffusion method for determination of colistin susceptibility is unreliable, which is attributed to the poor and unpredictable diffusion of colistin in the agar [21,22]. Therefore, in our study, we used the broth microdilution method for the determination of the minimal inhibitory concentration (MIC) to test for the colistin susceptibility. Broth microdilution is recommended by both EUCAST as well as CLSI for testing colistin susceptibility. Strains that show colistin minimal inhibitory concentrations (MICs) > 2 mg/L were interpreted as resistant according to the EUCAST breakpoints [23].

### 2.5. Detection of mcr Genes by Multiplex PCR

*E. coli* isolates that showed phenotypic resistance to colistin by the broth microdilution method were screened to detect *mcr-1, mcr-2, mcr-3, mcr-4*, and *mcr-5* genes by multiplex PCR (mPCR) according to the method described by Rebelo et al. [10]. Bacterial DNA was isolated from overnight cultures in Luria–Bertani medium using the Qiagen DNeasy DNA Extraction Kit (Qiagen, Hilden, Germany) according to the manufacturer’s instructions. The PCR reaction consisted of 12.5 μL 2 × MyTaq Red Mix (Bioline, London, UK), 0.2 μM of each primer, and 2 μL bacterial DNA. The sequences of the primers and the size of the amplified target products are listed in Table 1. The PCR conditions were: 1 cycle of initial denaturation at 95 °C for 10 min, followed by 30 cycles of initial denaturation at 94 °C for 30 s, an annealing step at 58 °C for 90 s, and an elongation step at 72 °C for 90 s, then a final elongation step at 72 °C for 10 min. Synthetic DNA fragments were used as controls. The identity of the amplified amplicons was confirmed by sequencing.

### 2.6. Detection of Extended-Spectrum β-Lactamase and Metallo β-Lactamase Production

*E. coli* isolates were screened for production of extended-spectrum β-lactamase (ESBL) by testing for reduced sensitivity to one or more of ceftriaxone, cefotaxime, and ceftazidime. These potential ESBL producers were confirmed by the modified double-disc synergy test as previously described [24]. The organism was considered an ESBL producer when an increase in the zone of inhibition of cefotaxime, ceftriaxone, and cefepime towards a disc containing amoxicillin clavulanate was observed. In addition, in the presence of clavulanic acid, the MIC values for ceftazidime and cefotaxime were lowered by ≥3 twofold dilutions as tested by the E-test [24]. Moreover, *E. coli* isolates were screened for metallo β-lactamase (MBL) production by testing for reduced susceptibility to imipenem or meropenem. Suspected strains were considered MBL producers when the inhibition zone diameters were enhanced in the presence of EDTA as tested by the combined disc assay using imipenem and imipenem/EDTA discs [25]. To evaluate the molecular basis of the resistance phenotypes, we assayed for the common ESBL (*bla_TEM-type_, bla_CTX-M-type_,* and *bla_SHV-type_*) or common MBL (*bla_IMP-type_,* and *bla_NDM-1_*) genes by PCR with specific primers (Table 1).

### 2.7. Identification of Different E. coli Pathotypes by mPCR

The isolates were screened for the presence of different virulence genes (*uidA, escV, bfpB, stx1, stx2, elt, estIa, estIb, invE, astA, aggR,* and *pic*) by the mPCR method using the same technique described by Muller et al. [26]. Reactions were performed in a total volume of 25 μL, containing 0.25 μL of Platinum Taq DNA polymerase (5 U/μL; Thermo Scientific, Waltham, MA, USA), 84 mM Tris HCl, 50 mM KCl, 2.1 mM MgCl_2_, 14 mM 2-mercaptoethanol, and 0.3 mM of each dNTP. Reaction conditions included an initial denaturation at 94 °C for 5 min, followed by 35 cycles of 94 °C for 30 s (denaturation), 63 °C for 30 s (annealing), and 72 °C for 1.5 min (extension), then a final extension for 5 min at 72 °C. Amplified products were visualized using 1.5% agarose gel electrophoresis. Primers used in the multiplex reactions are shown in Table 1. *E. coli* isolates were differentially classified into different pathotypes according to the profile of the tested virulence genes as described previously [26]. Enteropathogenic *E. coli* (EPEC) were identified as *escV*^+^ *bfp*^+^ *stx^−^*, Shiga toxin-producing *E. coli* (STEC) as *escV^+^*^/*−*^ *bfp^-^ stx^+^*, enterotoxigenic *E. coli* (ETEC) as *elt^+^* and/or *estIa^+^ estIb^+^*, enteroinvasive *E. coli* (EIEC) as *invE^+^*, enteroaggregative *E. coli* (EAEC) as *astA*^+^ and/or *aggR^+^* and/or *pic^+^*, and atypical *E. coli* (ATEC) as *escV*^+^ *bfp^−^ stx*^−^.

**Table 1:** Primers used for the multiplex PCR detection of *mcr1* to *mcr5*, *E. coli* virulence-related genes, and β-lactamase enzymes.

Target Gene	Sequence (5′–3′)	Amplified Product (bp)	Reference
*mcr-1*	AGTCCGTTTGTTCTTGTGGC AGATCCTTGGTCTCGGCTTG	320	[10]
*mcr-2*	CAAGTGTGTTGGTCGCAGTT TCTAGCCCGACAAGCATACC	715	
*mcr-3*	AAATAAAAATTGTTCCGCTTATG AATGGAGATCCCCGTTTTT	929	
*mcr-4*	TCACTTTCATCACTGCGTTG TTGGTCCATGACTACCAATG	1116	
*mcr-5*	ATGCGGTTGTCTGCATTTATC TCATTGTGGTTGTCCTTTTCTG	1644	[27]
EPEC, ATEC, STEC *escV*	ATTCTGGCTCTCTTCTTCTTTATGGCTG CGTCCCCTTTTACAAACTTCATCGC	544	[26]
EPEC *bfpB*	GACACCTCATTGCTGAAGTCG CCAGAACACCTCCGTTATGC	910	
STEC *stx1*	CGATGTTACGGTTTGTTACTGTGACAGC AATGCCACGCTTCCCAGAATTG	244	
STEC *stx2*	GTTTTGACCATCTTCGTCTGATTATTGAG AGCGTAAGGCTTCTGCTGTGAC	324	
ETEC *elt*	GAACAGGAGGTTTCTGCGTTAGGTG CTTTCAATGGCTTTTTTTTGGGAGTC	655	
ETEC *estIa*	CCTCTTTTAGYCAGACARCTGAATCASTG CAGGCAGGATTACAACAAAGTTCACAG	157	
ETEC *estIb*	TGTCTTTTTCACCTTTCGCTC CGGTACAAGCAGGATTACAACAC	171	
EIEC *invE*	CGATAGATGGCGAGAAATTATATCCCG CGATCAAGAATCCCTAACAGAAGAATCAC	766	
EAEC *astA*	TGCCATCAACACAGTATATCCG ACGGCTTTGTAGTCCTTCCAT	102	
EAEC *aggR*	ACGCAGAGTTGCCTGATAAAC AATACAGAATCGTCAGCATCAGC	400	
EAEC *pic*	AGCCGTTTCCGCAGAAGCC AAATGTCAGTGAACCGACGATTGG	1111	
*uidA*	ATGCCAGTCCAGCGTTTTTGC AAAGTGTGGGTCAATAATCAGGAAGTG	1487	
*bla_IMP_*	GGAATAGAGTGGCTTAAYTCTC GGTTTAAYAAAACAACCACC	232	[28]
*bla_NDM-1_*	CAGCGCAGCTTGTCG TCGCGAAGCTGAGCA	784	[29]
*bla_TEM_*	ATGAGTATTCAACATTTCCGTG TTACCAATGCTTAATCAGTGA	861	[30]
*bla_SHV_*	TTATCTCCCTGTTAGCCACC GATTTGCTGATTTCGCTCGG	795	[31]
*bla_CTX-M_*	SCSATGTGCAGYACCAGTAA CCGCRATATGRTTGGTGGTG	544	[32]

### 2.8. Statistical Analysis

The results are shown as frequencies (%) for qualitative variables. The normality of the data distribution was assessed by the Kolmogorov–Smirnov test. Comparisons between proportions were carried out using the chi-square test or Fisher’s exact test using Pearson’s probability value (*p*-value) where appropriate. Comparisons between quantitative variables were carried out by the Mann–Whitney test. SPSS software (SPSS 17.0, Chicago, IL, USA) was used for data analysis. A *p*-value less than 0.05 was considered statistically significant.

## 3. Results

A total of 2340 patients were admitted to chest ICU over the 36-month duration of the study, of whom 702 developed nosocomial infections (30.1%). There were 140/702 (19.9%) cases of nosocomial pneumonia infected with *E. coli*, of whom 76/140 (45.2%) were males and 64/140 (45.8%) were females.

Patients had a mean age of 54.5 ± 10.3 years and all of them had received empirical antibiotics, but none of them had been given colistin. Most of the patients (72.9%) required urinary catheters. Baseline characteristics of the enrolled patients are shown in Table 2.

As tested by broth microdilution, 21/140 (15%) isolates were resistant to colistin. Of note, the prevalence of colistin resistance increased over the study duration (3 years). Colistin resistance was observed in 5/55 (9.1%) patients during the first year of the study, and the rate of resistance increased to 8/49 (16.3%) patients in the next year and reached 8/36 (22.2%) patients in the third year of the study duration.

The MIC of the resistant isolates ranged from 4–16 mg/L: MIC_50_ was 4 mg/L, while MIC_90_ was 16 mg/L. Multiplex PCR screening for *mcr* genes (*mcr-1* to *mcr-5*) showed that all colistin-resistant isolates harbored *mcr-1*, and no other mobile colistin resistance gene was detected. There were no differences between the colistin-resistant and colistin-sensitive groups regarding sex, age, diabetes mellitus, obesity, malnutrition, urinary tract catheterization, central venous catheterization, artificial feeding, or the need for mechanical ventilation (Table 2), indicating that the presence of these risk factors was not associated with resistance to colistin.

All colistin-resistant *E. coli* isolates also showed co-resistance to amoxicillin and amoxicillin/clavulanic acid (21/21; 100%). Moreover, a high proportion of the isolates showed co-resistance to aztreonam (10/21; 47.6%), ceftriaxone (9/21; 42.9%), chloramphenicol (9/21; 42.9%), and ciprofloxacin (9/21; 42.9%) (Table 3). However, a similar trend was also observed among the colistin-sensitive isolates (*p* > 0.05), indicating that co-resistance to other antibiotics was not characteristic of the colistin-resistant strains.

Resistance to third-generation cephalosporins correlated with the rate of ESBL production, while resistance to carbapenems correlated with MBL production. Regarding ESBL production, 9 (42.9%) and 43 (36.1%) of colistin-resistant and colistin-sensitive groups were ESBL producers, while 5 (23.8%) and 25 (21%) were MBL producers, respectively. *bla_TEM-type_* was the most frequently detected ESBL gene, while *bla_IMP-type_* was the most common MBL in both groups (Table 3). In the colistin-resistant isolates, two strains carried *bla_NDM_*+ *bla_TEM-type_*, and one strain carried *bla_IMP-type_* + *bla_CTX-M-type_*. However, in the colistin-sensitive group, six isolates had *bla_NDM_* + *bla_TEM-type_*, two isolates had *bla_IMP-type_* + *bla_CTX-M-type_*, and two isolates had *bla_IMP-type_* + *bla_NDM_* + *bla_TEM-type_*.

Overall, the isolated *E. coli* strains showed a high frequency of virulence genes. The genes *aggR, escV*, and *astA* showed the highest frequencies of 25.7%, 25%, and 21.4%, respectively. Other genes were also observed but at a lower rate (Table 4). Interestingly, colistin resistance was statistically correlated with *astA, aggR*, and *pic* genes (*p* = 0.001, 0.002, 0.001, respectively).

Moreover, we classified the clinical *E. coli* isolates according to their pathotypes (Table 5), and the most common detected *E. coli* strains were enteroaggregative *E. coli* (EAEC), followed by enterotoxigenic *E. coli* (ETEC) and atypical *E. coli* (ATEC) (rates of detection = 25.7%, 23.6%, and 20.7%, respectively). Importantly, colistin resistance was statistically associated with the enteroaggregative *E. coli* (EAEC) samples (76.2%; *p* = 0.0001). Lower levels of colistin resistance were observed among enteropathogenic *E. coli* (EPEC) (9.5%; *p* = 0.059) and Shiga toxin-producing *E. coli* (STEC) (9.5%; *p* = 0.059).

## 4. Discussion

Despite the improvements in therapeutic agents and infection control practice, hospital-acquired pneumonia is still a major health problem. Nosocomial pneumonia is a leading cause of morbidity and mortality and is associated with longer hospital stays (7–9 days), particularly when coupled with antibiotic-resistant organisms [33]. The Center for Disease Control and Prevention (CDC) and the Nosocomial Infection Surveillance System (NISS) have reported that the most important causative agents for nosocomial pneumonia are the Gram-negative bacilli [34,35].

Patients admitted to chest intensive care units are frequently subjected to prolonged antibiotic therapy, which increases the risk of the development of antibiotic resistance, which explains the high rates of antimicrobial resistance observed in our samples [36,37,38,39,40,41]. The increased resistance to multiple antibiotics in Gram-negative bacteria presents significant global health-care problems because these MDR Gram-negative pathogens are often not controlled by the available and effective antibiotics [42,43,44]. The advent of MDR Gram-negative bacteria in recent years has made colistin an effective therapeutic alternative for various infections [45]. Two types of risk factor have been described for nosocomial pneumonia: the patient and factors related to infection prevention. Patient-related factors include prolonged hospitalization, malnutrition, acidosis, smoking, and the presence of comorbidities (CNS disease, chronic obstructive pulmonary disease, respiratory insufficiency, and diabetes mellitus) [18,46,47]. However, risk factors related to infection prevention include bad hygiene, failure of sterilization techniques, or inappropriate prolonged antibiotic treatment [48,49]. In our study, colistin resistance was not associated with age, sex, urinary tract catheters, diabetes mellitus, obesity, malnutrition, central venous catheters, artificial feeding, or laboratory parameters. However, the risk factors differed according to the geographical area and the analyzed population.

The present study described the prevalence of colistin resistance in clinical isolates of *E. coli* isolated from cases of nosocomial pneumonia. Colistin resistance was observed in 15% of the isolates, although none of the patients had received colistin. A similar high level of colistin resistance (23.1%) was observed in the same hospital among *E. coli* isolated from urine samples [50]. Overall, the rate of colistin resistance was higher when compared with other reported cases in other countries [51,52,53]. A study from the worldwide SENTRY antimicrobial surveillance program reported that resistance to polymyxins is stable for most Gram-negative pathogens except for *Klebsiella* species. *Pseudomonas aeruginosa (P. aeruginosa)*, *Acinetobacter* spp., and *Klebsiella* spp. showed resistance rates of 0.4%, 0.9%, and 1.5%, respectively [54]. In Nigeria and South Africa, colistin resistance rates were less than 10% [55]. Studies from Tunisia showed that 0.09% of *E. coli* and 1.2% of *Klebsiella pneumoniae* strains were resistant to colistin [56]. In the United Kingdom, a low percentage of *P. aeruginosa* strains were colistin-resistant (3.1%) [57]. A study from Spain showed that 19.1% of *Acinetobacter* *baumannii* (*A. baumannii)* strains were resistant to colistin [58]. Miftode et al. [59] analyzed the colistin resistance of *E. coli* and *Klebsiella* in Romania and reported 11% and 17% of them to be colistin-resistant, respectively. The use of empirical antibiotic therapy may lead to an increase in the detection of the resistant isolates because susceptible isolates may not be detected. However, adequate empirical antibiotics are essential to decrease the length of hospitalization and achieve better clinical resolution in both hospital and ventilator-associated cases of pneumonia [60].

Alarmingly, when we analyzed the prevalence of the colistin resistance over the 3 years (36 months), we noticed a steady increase in the resistance rates to colistin from 9.1% in the first year to 16.3% in the second year and 22.2% in the third year. Therefore, active surveillance of colistin resistance is very important in our university hospital to monitor and prevent the further spread of the nosocomial colistin-resistant strains. Colistin resistance was first identified in Egypt in *E. coli* isolated from a sputum sample from a patient admitted to ICU of Cairo City Hospital [61]. Interestingly, this isolate belonged to sequence type ST1011, which was described in Egypt as an avian fecal strain [62]. Another alarming rate for colistin resistance in Egypt was reported among cancer patients, where 8.8% of *Klebsiella pneumoniae (K. pneumoniae)* and *E. coli* strains were colistin-resistant [63]. In agreement with different reports, in our study, resistance to colistin was mediated by the presence of the *mcr-1* gene, which is responsible for the addition of phosphoethanolamine to the lipid A fraction of the cell wall [64,65,66]. However, we did not test the presence of the recently reported types of *mcr* such as *mcr 6–10* or the *pmrA/B* and *phoP/Q* genes. Importantly, Snesrud et al. [67] showed that *mcr-1* could be mobilized as a transposon which may reflect the wide spread of colistin resistance in the Gram-negative bacilli.

Of note, our isolates showed a high colistin resistance rate, although none of the patients had received colistin. This could be explained, in general, by the increased exposure and use of antimicrobials, leading to the selection of multidrug-resistant microorganisms and horizontal pathogen transmissions [68]. It is also possible that resistant strains have become more prevalent and widespread in the hospital environment, regardless of the particular risk factors of the group of patients involved, and are circulating in the hospital environment [69]. Importantly, a similar rate of colistin resistance was observed in the same hospital in *E. coli* strains isolated from the urine samples of patients suffering from urinary tract infections [50].

At the community level, colistin use in animal production was proposed as the most likely factor leading to the development of *mcr-1*. Liu et al. [7] showed that colistin usage in chicken farms was associated with increased colonization of chickens with *mcr-1* carrying bacteria. Furthermore, human exposure to *mcr-1*^+^ chickens was associated with increased colonization with colistin-resistant bacteria, pointing to the need to restrict the use of colistin as an antimicrobial drug in animals used for food production [70]. In Portugal, screening of 100 rectal swabs from pigs revealed 98 *mcr-1*-positive isolates [71]. It is possible that persistent exposure to low levels of colistin, such as those originating from the use of colistin-containing poultry food, led to the development of colistin resistance [64].

In fact, the actual regular rate of resistance to colistin is not known, as it is not tested on a routine in our microbiology laboratories. Colistin resistance is typically tested in MDR isolates when it is considered for treatment [66]. Generally, the tested *E. coli* strains showed an MDR phenotype regardless of colistin resistance.

Of the 140 *E. coli* isolates, 52/140 (37.1%) were ESBL producers and 30/140 (21.4%) were MBL producers, with the predominance of *bla_TEM-type_* and *bla_IMP-type_* resistance-associated genes, regardless of colistin resistance status, which is in line with previous studies from the university hospital [36,72]. Many isolates carried more than one resistance-associated gene. This high level of resistance is consistent with the fact that these isolates were nosocomial in origin, which are well-known for their resistance to multiple antibiotics. Bradford et al. [73] showed that colistin-resistant Enterobacteriaceae isolates showed a strong association with carrying ESBL enzymes such as *bla_CTX-M-type_, bla_SHV-type_*, and *bla_VEB_* variants, and/or a high level of KPC type carbapenemase (65.5%). Kontopoulou et al. [74] described a clonal spread of a colistin-resistant *K. pneumoniae* that produced a carbapenemase-type of β-lactamase in hospitals in Greece. In Tunisia, 7/29 carbapeneme-resistant *K. pneumoniae* were also resistant to colistin and were responsible for an outbreak in a university hospital [75].

Importantly, most of the colistin-resistant *E. coli* identified in this study also exhibited a high carriage rate of virulence genes: *astA* (76.2%), *aggR* (76.2%), and *pic* (52.4%). These genes are responsible for the production of enteroaggregative *E. coli* heat-stable toxin-1, a regulator for a number of virulence-associated genes, and a serine protease precursor, respectively [76]. These genes are characteristic of the enteroaggregative type of *E. coli*. Although EAEC in this study were isolated from pneumonia patients, these strains, in fact, commonly cause persistent diarrhea in children [77]. Extraintestinal *E. coli* infections emerged during the 2000s and are characterized by a high level of antibiotic resistance, especially to B-lactams and fluoroquinolones. The extraintestinal infections of *E. coli* included nosocomial pneumonia, bacteremia, and neonatal meningitis [78]. These isolates often exhibit a broad range of virulence-related genes, including toxins, adhesion, and invasion factors, which increase the ability to colonize the lungs and other body tissues [3,79].

*astA* is similar to the heat-stable enterotoxin ETEC and it functions by interfering with cGMP signaling, leading to secretion of anions and impaired fluid balance across the epithelium [80]. *AggR* is a transcriptional activator that stimulates the production of many plasmid- and chromosomal-mediated virulence genes [81]. Pic has diverse biological functions; it has a mucinolytic activity, which might enhance the ability of *E. coli* to penetrate the mucus layer and promote bacterial colonization [82]. It plays a potential role in complement resistance through protease activity [83]. Pic may also contribute to the production of biofilms [84].

Despite a huge amount of research accumulated on the mechanism of *E. coli*’s damage in gastro-intestinal tract, urinary tract, bloodstream, and CNS infections, there is, surprisingly, little data on pulmonary infections, although *E. coli* causes infections in ICUs as frequently as *Pseudomonas* [85]. The main *E coli* pathotypes detected in our cases were the enteroaggregative *E. coli,* which carry the *AggR* gene responsible for aggregative adherence to tissue. This type of *E. coli* is known to cause infection in elderly or immunocompromised patients, which is consistent with the fact that our patients are in the ICU. The role of the different *E. coli* virulence factors, including Shiga toxin, in pneumonia pathogenesis has not been clearly investigated, and we did not carry out experimental analysis on the role of the toxins in pulmonary cells. Increased awareness of the geno- and phenotypic characters of *E. coli* causing pneumonia in ICUs is a crucial first step in identifying preventive strategies. To the best of our knowledge, this is the first study of the correlation between colistin resistance in *E. coli* and the distribution of the virulence-related genes and *E. coli* pathotypes.

## 5. Conclusions

In conclusion, the high colistin resistance rate observed among *E. coli* strains isolated from patients with nosocomial pneumonia in our university hospital is worrisome. These isolates carry different drug resistance and virulence-related genes. Our results indicate that strict monitoring of colistin-resistant *E. coli* in our university hospital is necessary. Furthermore, infection control policies restricting the unnecessary use of extended-spectrum cephalosporins and carbapenems are needed.

## Figures and Tables

**Table 2:** Baseline characteristics of the patients admitted to the chest intensive care unit (ICU) with pneumonia.

Risk Factors	Total Patients Infected with *E. coli* (*n* = 140)	Colistin-Resistant Group (*n* = 21)	Colistin-Sensitive Group (*n* = 119)	*p*-Value
Sex (male)	76 (45.2%)	13 (61.9%)	63 (52.9%)	0.512
Age (years)	54.5 ± 10.3	57.6 ± 6.4	53.7 ± 10.9	0.872
Empiric antibiotic therapy during hospitalization	140 (100%)	21 (100%)	119 (100%)	NA
Urinary tract catheter	102 (72.9%)	17 (81.0%)	85 (71.4%)	0.366
Diabetes mellitus	51 (36.4%)	9 (42.9%)	42 (35.3%)	0.507
Obesity	42 (30.0 %)	6 (28.6%)	36 (30.3%)	0.877
Malnutrition	39 (27.9%)	6 (28.6%)	33 (27.7%)	0.937
Central venous catheter	13 (9.3%)	2 (9.5%)	11 (9.2%)	1.000
Artificial feeding	14 (10%)	1 (4.8%)	13 (10.9%)	0.694
Mechanical ventilation	22 (15.7%)	4 (19.0%)	18 (15.1%)	0.745
Worsening oxygenation	112 (80%)	15 (76.2)	96 (80.7%)	0.732
Pleural effusion	20 (14.3%)	3 (14.3%)	17 (14.3%)	0.979
Cavitation on chest radiograph	5 (3.5%)	1 (4.7%)	4 (3.4%)	0.891
WBC (×10^9^/L)	8.1 ± 3.7	7.6 ± 3.2	8.6 ± 3.4	0.547
Hemoglobin (g/dL)	12.6 ± 1.2	12.3 ± 1.6	12.9 ± 1	0.378
Platelets (×10^3^/μL)	277 ± 84	273 ± 80.1	281 ± 84	0.978

NA = Not applicable.

**Table 3:** Co-resistance profile and resistance-associated genes for *E. coli* isolates.

Antibiotic	Colistin-Resistant Group *n* = 21	Colistin-Sensitive Group *n* = 119	*p*-Value
Amikacin	5 (23.8%)	23 (19.3%)	0.767
Amoxicillin	21 (100%)	119 (100%)	NA
Amoxicillin/clavulanic acid	21 (100%)	119 (100%)	NA
Aztreonam	10 (47.6%)	70 (58.8%)	0.339
Cefaclor	7 (33.3%)	52 (43.7%)	0.375
Ceftriaxone	9 (42.9%)	43 (36.1%)	0.557
Cephazoline	6 (28.6%)	40 (33.6%)	0.650
Chloramphenicol	9 (42.9%)	57 (47.9%)	0.670
Ciprofloxacin	9 (42.9%)	49 (41.2%)	0.885
Levofloxacin	2 (9.5%)	23 (19.3%)	0.367
Meropenem	4 (19.0%)	24 (20.2%)	1.000
Imipenem	5 (23.8%)	29 (24.4%)	0.956
Resistance-associated genes			
*bla_TEM-type_*	5 (23.8%)	20 (16.8%)	0.124
*bla_CTX-M-type_*	2 (9.5%)	8 (6.7%)	0.792
*bla_SHV-type_*	0	0	NA^#^
*bla_IMP-type_*	3 (14.3%)	12 (10.1%)	0.467
*bla_NDM-1_*	2 (9.5%)	9 (7.6%)	0.669

NA = Not applicable.

**Table 4:** Distribution of virulence-related genes among *E. coli* isolates.

Gene	All *E. coli* Isolates *n* = 140	Colistin-Resistant *n* = 21	Colistin-Sensitive *n* = 119	*p*-Value
*escV*	35 (25%)	2 (9.5%)	33 (27.7%)	0.076
*bfpB*	3 (2.1%)	2 (9.5%)	1 (0.8%)	0.059
*stx1*	11 (7.9%)	2 (9.5%)	9 (7.6%)	0.670
*stx2*	7 (5%)	0 (0%)	7 (5.9%)	NA
*elt*	26 (18.6%)	0 (0%)	26 (21.8%)	NA
*estIa*	8 (5.7%)	0 (0%)	8 (6.7%)	NA
*estIb*	21 (15%)	0 (0%)	21 (17.6%)	NA
*invE*	13 (9.3%)	1 (4.8%)	12 (10.1%)	0.691
*astA*	30 (21.4%)	16 (76.2%)	14 (11.8%)	0.001 *
*aggR*	36 (25.7%)	16 (76.2%)	20 (16.8%)	0.002 *
*pic*	29 (20.7%)	11 (52.4%)	18 (15.1%)	0.001 *

* denotes significance with *p* < 0.05; NA = not applicable.

**Table 5:** Colistin resistance among the different *E. coli* pathotypes.

Type	All *E. coli* Isolates *n* = 140	Colistin-Resistant *n* = 21	Colistin-Sensitive *n* = 119	*p*-Value
Enteroaggregative *E. coli* (EAEC)	36 (25.7%)	16 (76.2%)	20 (16.8%)	0.0001 *
Atypical *E. coli* (ATEC)	29 (20.7%)	0 (0.0%)	29 (24.4%)	NA
Enterotoxigenic *E. coli* (ETEC)	33 (23.6%)	0 (0.0%)	33 (27.7%)	NA
Enteropathogenic *E. coli* (EPEC)	3 (2.1%)	2 (9.5%)	1 (0.8%)	0.059
Enteroinvasive *E. coli* (EIEC)	13 (9.3%)	1 (4.8%)	12 (10.1%)	0.691
Shiga toxin-producing *E. coli* (STEC)	18 (12.9%)	2 (9.5%)	16 (13.4%)	1.000

* denotes significance with *p* < 0.05; NA = not applicable.

## Data Availability

The data generated in this study are available from the corresponding author on reasonable request.
